# Insula network connectivity mediates the association between childhood maltreatment and depressive symptoms in major depressive disorder patients

**DOI:** 10.1038/s41398-022-01829-w

**Published:** 2022-03-02

**Authors:** Cancan He, Dandan Fan, Xinyi Liu, Qing Wang, Haisan Zhang, Hongxing Zhang, Zhijun Zhang, Chunming Xie

**Affiliations:** 1 Department of Neurology, Affiliated ZhongDa Hospital, School of Medicine, Southeast University, Nanjing, Jiangsu 210009 China; 2 Institute of Neuropsychiatry, Affiliated ZhongDa Hospital, Southeast University, Nanjing, Jiangsu 210009 China; 3 The Key Laboratory of Developmental Genes and Human Disease, Southeast University, Nanjing, Jiangsu 210096 China; 4 Department of Radiology, the Second Affiliated Hospital of Xinxiang Medical University, Xinxiang, Henan 453002 China; 5 Xinxiang Key Laboratory of Multimodal Brain Imaging, the Second Affiliated Hospital of Xinxiang Medical University, Xinxiang, Henan 453002 China; 6 Department of Psychiatry, the Second Affiliated Hospital of Xinxiang Medical University, Xinxiang, Henan 453002 China; 7 Psychology School of Xinxiang Medical University, Xinxiang, Henan 453003 China

**Keywords:** Depression, Diagnostic markers

## Abstract

Childhood maltreatment (CM) is a major risk factor for developing the major depressive disorder (MDD), however, the neurobiological mechanism linking CM and MDD remains unclear. We recruited 34 healthy controls (HCs) and 44 MDD patients to complete the childhood maltreatment experience assessment with Childhood Trauma Questionnaire (CTQ) and resting-state fMRI scan. Multivariate linear regression analysis was employed to identify the main effects of CM and depressive symptoms total and subfactors scores on bilateral anterior and posterior insula functional connectivity (IFC) networks, respectively. Mediation analysis was performed to investigate whether IFC strength mediates the association between CM and depressive symptoms. MDD patients showed significantly decreased connectivity in the dorsal medial prefrontal cortex and increased connectivity in the medial frontal gyrus in the bipartite IFC networks, compared to HCs. The main effects of CM and depressive symptoms showed a large discrepancy on the anterior and posterior IFC networks, which primarily located in the frontal-limbic system. Further, conjunction analysis identified the overlapping regions linking CM and depressive symptoms were mainly implicated in self-regulation and cognitive processing circuits. More important, these IFC strengths could mediate the association between different types of CM, especially for childhood abuse and childhood neglect, and depressive symptoms in those overlapping regions. We demonstrated that early exposure to CM may increase the vulnerability to depression by influencing brain’s self-regulating and cognitive processing circuitry. These findings provide new insight into the understanding of pathological mechanism underlying CM-induced depressive symptoms.

## Background

Major depressive disorder (MDD) is the most common psychiatric condition worldwide and rank the third leading cause of years lived with disability (YLDs) in the past decade [1]. However, despite strenuous efforts, the neurobiological basis of MDD remains poorly understood [2, 3]. As a heterogeneous internalizing disorders, MDD can be characterized by multiple symptoms: persistence of depressed mood, diminished of interest or pleasure, weight change, insomnia or hypersomnia, psychomotor retardation, fatigue or loss of energy, and disturbance in cognitive functions, such as attention and memory [4]. Accumulating evidence have identified that such heterogeneity are sourced from complex physiological and biological factors.

Childhood maltreatment (CM), as one of the essential aspects of environmental stress, occupies an important position in the risk factors of MDD. Specifically, there are five types of CM: emotional, physical, and sexual abuse, and emotional and physical neglect. It has been suggested that CM could predict unfavorable outcomes in patients with MDD, including earlier onset, treatment-resistant, higher recurrent rate, and severer symptoms [5]. There is an assumption that maltreated individuals may represent a distinct clinical phenotype of MDD [5, 6]. However, whether CM could influence the types of clinical symptoms and associate with the heterogeneity of MDD is not clear yet. Emerging evidence suggests that CM alters brain structures and functions to affect sensory systems [7], or network architectures and circuits involved in threat detection [8], emotional regulation, and reward anticipation [9, 10]. More specifically, brain areas implicated in the pathophysiology of MDD also exhibit overall changes in the context of CM. For instance, the reward-related ventral striatum activity were found negatively related to depressive symptoms and partially mediated the association between emotional neglect and subsequent depressive symptoms in adolescent [11]. Several studies also found that MDD patients with and without childhood neglect exhibited distinct functional connectivity patterns, only MDD with childhood neglect showed widespread reduction of functional connectivity strength in the prefrontal-limbic-thalamic-cerebellar circuitry [12]. And current evidence confirmed that the reduced hippocampus volume in MDD patients compared with healthy controls (HCs), was limited to maltreated MDD phenotypes only [13, 14]. These findings pointing to brain alterations as adverse consequence of CM might enhance the vulnerability of subject who suffer CM experience to depression. Nevertheless, the association between CM, especially different types of CM, and pathophysiological mechanism of depression remains unclear.

As a critical hub of the paralimbic system, the insula is anatomically connected to a wide range of cortical, limbic and paralimbic structures [15, 16], and functionally implicated in high-order cognition, emotional responses, and empathic processes [17, 18]. Evidences from neuroimaging studies have shown that insula dysfunction contributes a lot to altered functionalities of MDD patients [19, 20]. In addition, it is also demonstrated that adolescents with CM exposure displayed an increased activation of the whole insula [21], and severe CM subjects exhibited exaggerated responses to fast touch in the right posterior insular cortex [22]. A recent longitudinal study also found that both previous CM experiences and future depression relapse were associated with reduced baseline surface area of right insula, and insular surface area was shown to mediate the association between CM and future depression relapse [23]. Our recent study also demonstrated that the functional connectivity in prefrontal-limbic circuits to amygdala could mediate the relationship among CM experience, epigenetic factors, and the severity of depression in MDD patient, and the CM experience integrated with functional connectivity strength in the prefrontal-limbic system serve as a good potential biomarker for depression diagnoses [24]. However, previous research did not take into account the dysfunctional connectivity of the insula as a key variable between CM and MDD. Research is needed to determine whether the insula functional connectivity alterations observed in MDD is affected by CM. Besides, decades of studies have revealed that the complicated functions of the insula were taking effects by mapping onto distinct subregions, the anterior insula and the posterior insula. However, little is known regarding relationships between CM subtypes, different insula functional network architectures, and the potential contributions to depressive symptoms in MDD patients.

Therefore, we aimed to examine the potential association between CM, insula functional network, and different depressive symptoms in MDD patients using the resting-state functional connectivity (RSFC) approach. Given the different functions of insula subregions, we hypothesized that childhood abuse and neglect burden in MDD patients would significantly influence the connectivity pattern of the insula subregional networks and subsequently develop to depression, and that the association between CM subtypes and adult depressive symptoms could be mediated by the alterations of specific insula subregional connectivity.

## Materials and methods

### Participants and study approval

A total of 81 participants, including 34 HCs and 47 patients with MDD, were enrolled. Power analysis software (Hintze, J. NCSS, LLC. Kaysville, Utah, USA. www.ncss.com) was used to calculate the sample power for the clinical study. All subjects were Chinese Han population and right-handed. MDD patients were recruited through inpatients at the Department of Psychiatry in Henan Provincial Mental Hospital. HCs were enrolled through local community posting and media advertising. The ethics committee of Henan Provincial Mental Hospital Affiliated with Xinxiang Medical University approved this research protocol (approval ID: 2017-08), written informed consents were signed by all participants or their legally authorized representatives. Three MDD patients were excluded due to excessive head motion during functional magnetic resonance imaging (fMRI) scanning. Hence, thirty-four HCs and 44 MDD patients entered further analysis.

### Inclusion and exclusion criteria

MDD patients met the following criteria: (1) the diagnostic criteria for MDD using a Structured Clinical Interview by two neuropsychiatrists (CM Xie and HX Zhang) according to the Diagnostic Statistical Manual of Mental Disorder, Fifth Edition (DSM-V); (2) the scores larger than 8 which was measured by Hamilton Depression Rating Scale (HAMD-17); (3) drug naïve or drug free for longer than three weeks; (4) age between 18 and 55. HCs were required to have a HAMD-17 score ≤ 7. Exclusion criteria for all subjects included the following: (1) other major psychiatric disorders or neurodegenerative disease history; (2) substance abuse, head trauma, or loss of consciousness; and (3) contraindications to MRI scanning.

### Behavior measurements

All subjects underwent a comprehensive clinical assessment of their neurological and mental status, including HAMD-17 for depression severity and Hamilton Anxiety Scale (HAMA) for anxiety evaluation. We also counted the five subscales of the HAMD-17 including anxiety/somatization, weight, cognitive disturbance, retardation, and sleep disruption [25]. The Childhood Trauma Questionnaire (CTQ), a self-report scale provided screening for a history of childhood maltreatment, which can be subdivided into five subscales: emotional abuse, physical abuse, sexual abuse, physical neglect, and emotional neglect.

### MRI acquisition

Imaging was conducted on Siemens 3.0 T scanner (Munich, Germany). Briefly, T1-weighted anatomical scans and resting-state functional runs of approximately 8 min were acquired from all participants (240 time points, 2-second repetition time, 3mm^3^ voxels). Detailed parameters and scanning methods are described in the Supplementary Materials.

### MRI data processing

The functional data were preprocessed using the DPABI toolbox (Data Processing and Analysis for Brain Imaging) (http:// rfmri.org/dpabi) [26], SPM12 toolkit (http://www.fil.ion.ucl.ac.uk/spm) and MATLAB version 2015b (The MathWorks, Inc., Natick, MA, USA). As a quality control metric, subjects with exceeded motion thresholds (>2.5 mm translational movement or >3° rotational movement) were discarded, and scans with mean framewise displacement (FD) > 0.2 mm were also excluded [27]. Data were normalized using the DARTEL toolbox [28] into Montreal Neurological Institute (MNI) space and smoothed with an 6-mm full-width half-maximum kernel. To further reduce the effects of confounding factors, all data were preprocessed to remove 24 motion parameter [29], cerebrospinal fluid signals, white matter signals, the global mean signal, and an overall linear trend. A bandpass filter was applied (0.01–0.08 Hz). Please see Supplementary Materials for more details.

### Voxel-wise based insula functional connectivity construction

Then, a voxel-wise, whole-brain functional connectivity analysis was conducted using SPM12. We created 4 spherical regions of interest (ROIs) of 6 mm radius for bilateral insula, included bilateral anterior insula (AI) ([–30, 20, 6] and [30, 26, 4]) and bilateral posterior insula ([–40, 4, –4] and [42, –6, –8]), to obtain the anterior and posterior insula functional connectivity (IFC) map for each participant [30]. The average time course in each ROI region, as the seed time course, was correlated with the time courses from all brain voxels using Pearson’s correlation, then Pearson’s coefficients were z-transformed. Thus, each individual IFC network map was obtained.

### Statistical analysis

#### Demographic and behavior data

Kolmogorov-Smirnov Test was used to assess normally distribution of each group (*p* > 0.05), and all data were normally distributed. F-tests were used for homogeneity of variance and all statistically compared groups are with similar variance (*p* > 0.05). The demographic and behavioral data comparisons between the two groups were tested using independent sample *t*-tests and Chi-square tests (for gender). All the data are presented as the means ± standard deviation (SD). The significance level was set at *P* < 0.05 (SPSS 25.0; SPSS Inc., Chicago, IL). Multiple comparison correction was conducted using Bonferroni correction.

### Group-level differences of bilateral anterior and posterior IFC networks

The imaging data statistical analysis was performed using the Analysis of Functional NeuroImage (AFNI) software (http://afni.nimh.nih.gov/afni). First, a random-effects one-sample *t-*test was used to obtain bilateral anterior and posterior IFC networks pattern in each group (Supplementary Materials). To examine the group difference of the anterior and posterior IFC network connectivity between MDD patients and HCs, a 2 × 2 ANOVA (group × side) analysis was performed (3dRegAna, AFNI). The voxel-wised significant threshold was set at *p* < 0.001, corrected for multiple comparisons at the cluster level with the latest version of the 3dClustSim program in AFNI_16.3.00 (gray matter mask correction (67,541 voxels), voxel level *p* < 0.01, cluster level α < 0.05, κ > 220, cluster size > 5940 mm^3^; https://afni.nimh.nih.gov/pub/dist/doc/program_help/3dClustSim. html).

### Behavioral significance of IFC networks

Multivariate linear regression analysis was employed to explore the behavioral significance of the IFC networks (3dRegAna, AFNI). The statistical threshold was set at *p* < 0.01 (3dClustSim corrected, cluster level α < 0.05, κ > 220, cluster size > 5940 mm^3^). The multivariate linear regression analysis shown in the following equation was employed [31]:

$$m_i = \alpha _0 + \alpha _1 \ast CTQ + \alpha _2 \ast Hemi + \alpha _3 \ast Gen + \alpha _4 \ast Edu + \alpha _5 \ast Age + \alpha _6 \ast GMV + \varepsilon$$

where m_*i*_ is the m value of ith voxel across group subjects, *α*_0_ is the intercept of the straight-line fitting of the model, *α*_1_ is the main effect of the CTQ scores of the functional connectivity strength of each voxel in the IFC networks in MDD patients, and *α*_2_, *α*_3_, *α*_4_, *α*_5_ and *α*_6_ are the main effects of the hemisphere, gender, education, age, and GM volume covariates, respectively. The error term ε is assumed to have a Gaussian distribution and to be uncorrelated across subjects. Using this approach, we separately calculated the significant effects of CTQ and HAMD-17 total and subscale scores on the bilateral anterior and posterior IFC networks in MDD patients.

Second, conjunction analysis (3dcalc, AFNI) was used to obtain the overlapping regions between every pair of the CTQ and HAMD-17 subfactors on the bilateral anterior and posterior IFC networks.

### Mediation analysis

Given the significant influence of CTQ scores and depressive symptoms on IFC strength observed in MDD patients, mediation analyses were conducted to test the theoretical indirect association between childhood trauma and depressive symptoms, via anterior and posterior insula connectivity (SPSS 25.0; SPSS Inc., Chicago, IL). An indirect effect was considered significant if 95% bias-corrected confidence intervals (CI) from bootstrapped analyses (10,000 resamples) did not contain zero. This approach is based on a standard three-variable mediation model and is in line with the currently most widely accepted mediation analysis technique [24, 32]. A detailed description is provided in Supplementary Materials.

## Results

### Demographic and behavioral data

Demographic and clinical characteristics of the study are presented in Table 1. No significant differences were found in age and gender between the HCs and MDD patients, while the education years of MDD patients were significantly lower than those of HCs. MDD patients showed significantly higher scores of HAMD-17 and CTQ total and subscale, as well as higher HAMA scores. To avoid false-positive rate, the different significance of clinical characteristics were tested with *p* < 0.05/13 = 0.0038 (Bonferroni correction).

**Table 1:** Demographic and neuropsychiatric characteristics of MDD patients and HC subjects.

	HC (*N* = 34)	MDD (*N* = 44)	*p*
**Demographic characteristics**			
Age (years)	34.44 ± 11.76	39.02 ± 12.24	0.050
Gender (Male/Female)	12/22	23/21	0.138^†^
Education (years)	13.82 ± 3.09	10.23 ± 3.33	0.001
**Clinical characteristics**			
HAMD-17	1.21 ± 1.36	16.11 ± 5.88	0.001
HAMD-Anxiety	0.62 ± 0.85	4.73 ± 2.24	0.001
HAMD-Weight	0.00 ± 0.00	0.33 ± 0.69	0.001
HAMD-Cognition	0.11 ± 0.33	2.55 ± 1.54	0.001
HAMD-Retardation	0.24 ± 0.50	6.55 ± 2.72	0.001
HAMD-Sleep	0.18 ± 0.39	3.80 ± 2.28	0.001
CTQ	33.67 ± 7.28	45.07 ± 11.13	0.001
CTQ-PA	5.56 ± 1.19	6.84 ± 2.50	0.002
CTQ-EA	6.53 ± 1.93	8.36 ± 3.56	0.006
CTQ-SA	5.41 ± 0.89	6.36 ± 2.29	0.015
CTQ-PN	7.32 ± 2.70	10.36 ± 3.20	0.001
CTQ-EN	8.82 ± 3.67	13.27 ± 5.10	0.001
HAMA	1.26 ± 1.69	16.11 ± 5.88	0.001
Total disease duration (months)	NA	72.39 ± 81.30	NA
Current disease duration (months)	NA	5.70 ± 6.02	NA
Suicidal ideation (yes/no)	NA	31/13	NA
Family history (with/without)	NA	10/34	NA

*HC* Healthy Control, *MDD* Major Depressive Disorder, *HAMD-17* 17-items Hamilton Depression Scale, *HAMD-Anxiety* HAMD-17 Anxiety/somatization subscale, *HAMD-Weight* HAMD-17 Weight loss subscale, *HAMD-Cognition* HAMD-17 Cognitive disturbance subscale, *HAMD-Retardation* HAMD-17 Retardation subscale, *HAMD-Sleep* HAMD-17 Sleep disruption subscale, *CTQ* Childhood Trauma Questionnaire, *CTQ-PA* CTQ Physical Abuse subscale, *CTQ-EA* CTQ Emotional Abuse subscale, *CTQ-SA* CTQ Sexual Abuse subscale, *CTQ-PN* CTQ Physical Neglect subscale, *CTQ-EN* CTQ Emotional Neglect subscale, *HAMA* Hamilton Anxiety Scale, *NA* Not available.

^†^*p* value was obtained by chi-square test; other *p* values were obtained by two-sample *t*-test between all MDD patients and HC subjects. Unless otherwise indicated, data are presented as the mean ± standard deviation.

### Group-level comparison of anterior and posterior IFC networks

**Fig. 1:**
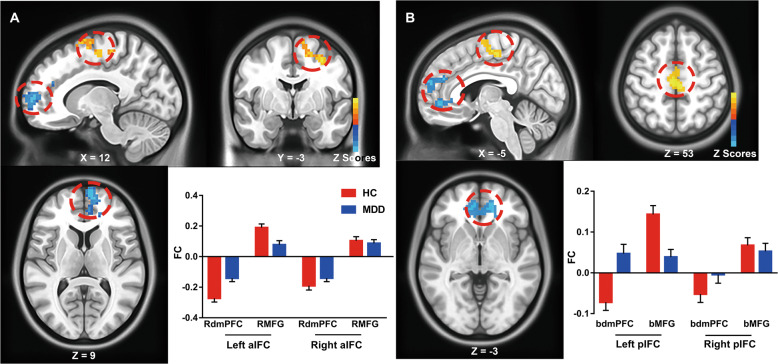
Hypo- and hyper-connectivity of anterior and posterior insula functional connectivity (IFC) networks between MDD patients and HC subjects. The results illustrate the differential functional connectivity of the anterior IFC (aIFC) **(A)** and posterior IFC (pIFC) **(B)** networks in MDD patients compared with HC subjects. Red color indicates hyperconnectivity and blue color indicates hypoconnectivity. Numerical representation of differential functional connectivity in specific regions was also shown with histogram. aIF, anterior insula functional networks, pIFC posterior insula functional networks, RdmPFC right dorsal medial prefrontal cortex, RMFG right medial frontal gyrus, bdmPFC bilateral dorsal medial prefrontal cortex, bMFG bilateral medial frontal gyrus.

The bilateral anterior IFC (aIFC) and posterior IFC (pIFC) patterns of the HCs and MDD patients are briefly illustrated in Fig. S1. Each IFC network was composed of both a positive network, which primarily located in medial PFC and subcortical regions, and a negative network, which mainly observed in the PFC-parietal system. Then, we examined the group differences of connectivity specificity in the bilateral aIFC and pIFC networks. As shown in Fig. 1, several regions consistently showed altered connectivity in MDD patients. Specifically, MDD patients represented decreased functional connectivity right dorsal medial prefrontal cortex (dmPFC) and increased functional connectivity in right medial frontal gyrus (MFG) within the aIFC network, while decreased connectivity in bilateral dmPFC and increased connectivity in bilateral MFG within the pIFC network, compared to HCs.

### Main effects of CM on IFC networks in MDD patients

**Fig. 2:**
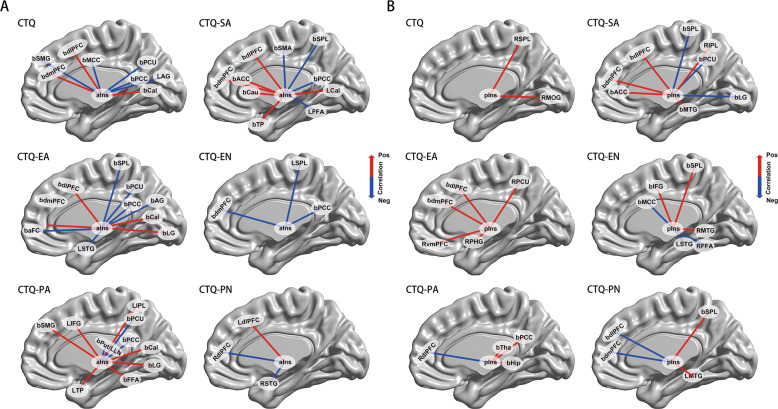
Neural effects of CTQ total and subscales scores on the bilateral aIFC and pIFC networks in MDD patients. Main effects of CTQ scores, CTQ-SA scores, CTQ-EA scores, CTQ-EN scores, CTQ-PA scores, and CTQ-PN scores on the bilateral aIFC networks (**A**) and pIFC networks (**B**) in the MDD patients. Red color indicates positive correlation and blue color indicates negative correlation. CTQ Childhood Trauma Questionnaire, CTQ-PA CTQ Physical Abuse subscale, CTQ-EA CTQ Emotional Abuse subscale, CTQ-SA CTQ Sexual Abuse subscale, CTQ-PN CTQ Physical Neglect subscale, CTQ-EN CTQ Emotional Neglect subscale, aIns anterior insula, pIns posterior insula, bSMG bilateral supramarginal gyrus, bdlPFC bilateral dorsal lateral prefrontal cortex, bdmPFC bilateral dorsal medial prefrontal cortex, bMCC bilateral middle cingulate cortex, bPcu bilateral precuneus, bPCC bilateral posterior cingulate cortex, LAG left angular gyrus, bCal bilateral calcarine gyrus, bACC bilateral anterior cingulate cortex, bSMA bilateral supplementary motor area, bCau bilateral caudate nucleus, bSPL bilateral superior parietal lobe, LCal left calcarine gyrus, LFFA left fusiform area, bTP bilateral temporal pole, baFC bilateral anterior prefrontal cortex, bAG bilateral angular gyrus, bLG bilateral lingual gyrus, LSTG left superior temporal gyrus, LSPL left superior parietal lobe, LIFG left inferior frontal gyrus, LIPL left inferior parietal lobe, bFFA bilateral fusiform area, LTP left temporal pole, RdlPFC right dorsal lateral prefrontal cortex, LdlPFC left dorsal lateral prefrontal cortex, RSTG right superior temporal gyrus, RMOG right middle occipital lobe, RIPL right inferior parietal lobe, RSPL right superior parietal lobe, bMTG bilateral middle temporal gyrus, RvmPFC right ventral medial prefrontal cortex, RPcu right precuneus, RPHG right parahippocampus, bIFG bilateral inferior frontal gyrus, RMTG right middle temporal gyrus, RFFA right fusiform area, bHip bilateral hippocampus, bTha bilateral thalamus, LMTG left middle temporal gyrus.

The main effects of childhood maltreatment on IFC networks were detected by CTQ total and subscale scores, respectively, as shown in Fig. 2. First, the main effects of CTQ showed a large discrepancy between aIFC and pIFC networks. The main effect of CTQ scores on aIFC network mainly located in wide regions of the frontal lobe, including bilateral supramarginal gyrus (SMG), dmPFC, dorsal lateral prefrontal cortex (dlPFC) and middle cingulate cortex (MCC), and occipital lobe, including bilateral precuneus (Pcu), PCC, calcarine gyrus (Cal) and left angular gyrus (AG) (Fig. 2A). While the main effect of CTQ on the pIFC network were solely located in the right superior parietal lobe (SPL) and middle occipital lobe (MOG) (Fig. 2B). Second, the neural effects of CTQ subscale scores also varied largely between aIFC and pIFC networks. CTQ emotional abuse subscale (CTQ-EA) showed both positive and negative effect on aIFC network, while only positive effect existed on pIFC network. Moreover, there was only negative effect of CTQ emotional neglect subscale (CTQ-EN) on aIFC network and both positive and negative effect showed on pIFC network. Third, we found the CTQ subscale had wider influence on aIFC network than pIFC network. For example, the neural substrates of CTQ physical abuse subscale (CTQ-PA) on the aIFC network were mainly observed in wide range of frontal-occipital-temporal system and the limbic system, while on the pIFC network it were just located on partials regions as right dlPFC, and bilateral thalamus (Tha), PCC and hippocampus (Hip).

### Overlapping effects of CM and depressive symptoms on IFC networks in MDD patients

**Fig. 3:**
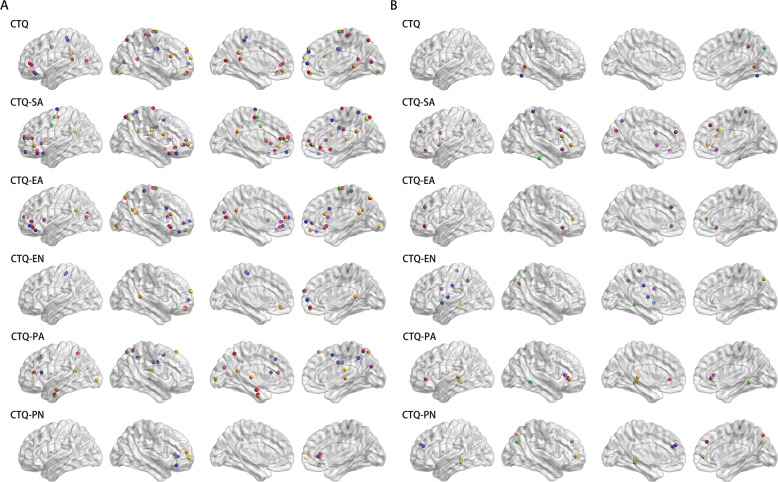
Overlapping regions of the main effects of CTQ total and subscales scores and HAMD-17 total and subscales scores on anterior and posterior IFC networks in MDD patients. **A** Overlapping effects between CTQ total and sub-factors and HAMD-17 total and sub-factors on the aIFC network. **B** Overlapping effects between CTQ total and sub-factors and HAMD-17 total and subfactors on pIFC network. Red nodes represent overlapping effects of HAMD-17; yellow nodes represent overlapping effects of HAMD-Weight; green nodes represent overlapping effects of HAMD-Sleep; orange nodes represent overlapping effects of HAMD-Anxiety; blue nodes represent overlapping effects of HAMD-Cognition; purple nodes represent overlapping effects of HAMD-Retardation. **Abbreviation:** aIFC anterior insula functional connectivity, pIFC posterior insula functional connectivity, CTQ Childhood Trauma Questionnaire, CTQ-PA CTQ Physical Abuse subscale, CTQ-EA CTQ Emotional Abuse subscale, CTQ-SA CTQ Sexual Abuse subscale, CTQ-PN CTQ Physical Neglect subscale, CTQ-EN CTQ Emotional Neglect subscale, HAMD-17 17-item Hamilton Depression Scale, HAMD-17 17-items Hamilton Depression Scale, HAMD-Anxiety HAMD-17 Anxiety/somatization subscale, HAMD-Weight HAMD-17 Weight loss subscale, HAMD-Cognition HAMD-17 Cognitive disturbance subscale, HAMD-Retardation HAMD-17 Retardation subscale, HAMD-Sleep HAMD-17 Sleep disruption subscale.

Conjunction analysis found the overlapping regions of CM and depressive symptoms on the bilateral IFC networks. As shown in Fig. 3, the aIFC network reported significantly more overlapping effect than the pIFC network in MDD patients. There was a clear trend that in the aIFC network, the CTQ abuse subscales (sexual, physical and emotional abuse), seemed to have greater interactions with depressive symptoms than CTQ neglect subscales (physical and emotional neglect). While in the pIFC network, the trend was not obvious. Interestingly, despite the distribution characteristics of CTQ total and subscale scores appeared somewhat different from one another, but there were still some common regions, especially for the frontal lobe, including bilateral dlPFC, dmPFC, and ventral medial prefrontal cortex (vmPFC), and the parietal lobe, including bilateral inferior parietal lobe (IPL), the cingulate cortex, as well as the subcortex regions, including Tha, caudate nucleus (Cau), Hip and parahippocampus gyrus (PHG). These findings represented the symptom-specific distribution of CM and depressive dimension on the bilateral insula networks in MDD patients. The detailed information was described in Table S2 and Table S3.

### Mediation analysis results

**Fig. 4:**
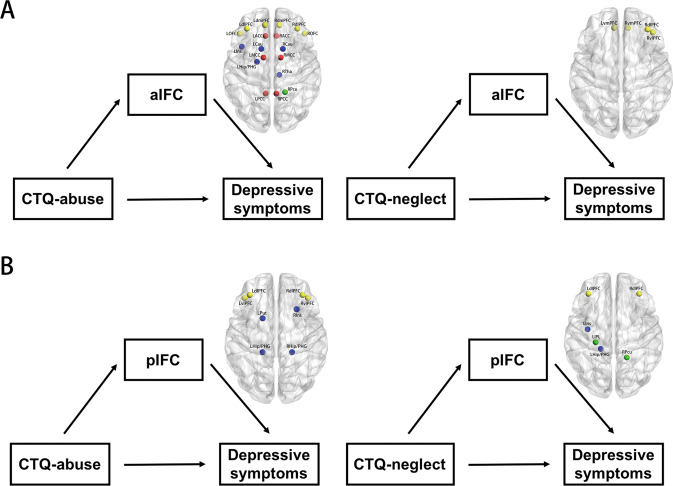
Mediation analysis revealed that different nodes within aIFC and pIFC could mediate the effects of childhood abuse and neglect on depressive symptoms. **A** Connectivity strength in brain regions within aIFC network mediates the association between childhood abuse and neglect and depressive symptoms. **B** Connectivity strength in brain regions within pIFC network mediates the association between childhood abuse and neglect and depressive symptoms. Yellow nodes represent the mediating nodes located in the prefrontal cortex; red nodes represent the mediating nodes located in the cingulate cortex; green nodes represent the mediating nodes located in the parietal lobe; blue nodes represent the mediating nodes located in the limbic/paralimbic system. **Abbreviation:** aIFC anterior insula functional connectivity, pIFC posterior insula functional connectivity, CTQ Childhood Trauma Questionnaire, LdlPFC left dorsal lateral prefrontal cortex, RdlPFC right dorsal lateral prefrontal cortex, LdmPFC left dorsal medial prefrontal cortex, RdmPFC right dorsal medial prefrontal cortex, LOFC left orbitofrontal cortex, ROFC right orbitofrontal cortex LACC left anterior cingulate cortex, RACC right anterior cingulate cortex, LMCC left middle cingulate cortex, RMCC right middle cingulate cortex, LPCC left posterior cingulate cortex, RPCC right posterior cingulate cortex, LCau left caudate nucleus, RCau right caudate nucleus, LIns left insula, LHip/PHG left hippocampus/parahippocampus, RTha right thalamus, RPcu right precuneus, LvmPFC left ventral medial prefrontal cortex, RvmPFC right ventral medial prefrontal cortex, RvlPFC, right ventral lateral prefrontal cortex, LvlPFC left ventral lateral prefrontal cortex, LPut left putamen, RIns right insula, RHip/PHG right hippocampus/parahippocampus, LIPL left inferior parietal lobe.

The results obtained from the exploratory mediation analysis are summarized in Fig. 4. It revealed a significant indirect effect of the IFC on the relationship between CM and depressive symptoms. In the aIFC network, the functional connectivity of bilateral dmPFC, dlPFC, orbitofrontal cortex (OFC), ACC, MCC, PCC, and Cau, left Hip/PHG and insula, right Tha and Pcu, could mediate the effects of childhood abuse experience on depressive symptoms. Whereas only few regions involved in the mediation of the association between childhood neglect experience and depressive symptoms, including bilateral vmPFC, right dlPFC and ventral lateral prefrontal cortex (vlPFC) (Fig. 4A). In the pIFC network, the functional connectivity of bilateral dlPFC, vlPFC, and Hip/PHG, left putamen (Put), and right Ins, could mediate the effects of childhood abuse experience on depressive symptoms, while bilateral dlPFC, left insula, IPL, Hip/PHG, and right Pcu, could mediate the effects of childhood neglect experience on depressive symptoms (Fig. 4B). The detailed information was described in Table S4.

## Discussion

To our knowledge, this study provides the first empirical evidence linking CM subtypes and depressive symptoms on the insula network connectivity in the MDD patients, and strongly supports our hypothesis that insula network could mediate the prospective association between early life stress and future depression. Our results identified that the neural effects of CM experience on IFC networks varied depending on the different insula subregional networks, and represented the symptom-specific distribution across different CM subtypes. Furthermore, the effects of different subtypes of CM on depressive symptoms, specifically childhood abuse and neglect, were mediated by the connectivity strength in distinctive regions connecting with different insula subregions.

In the current study, disruptions of the insula networks were found in MDD patients compared to HCs, which primarily located in the bilateral dmPFC and MFG. In accordance with the present results, previous resting-state fMRI researches have also shown that the insula exhibited reduced activity [33] and disrupted functional connectivity with salience network in unmedicated and treatment-resistant MDD patients [20]. Meanwhile, task-state fMRI study in MDD found the decreased functional connectivity of the insula during the interoceptive attention task [34]. Prior studies have noted the importance of the insula networks in transmitting the homeostatic information to subjective feelings and playing the crucial role in the neural circuits of emotional awareness [35, 36]. Our results add to the growing body of evidence that the disrupted insula network connectivity in MDD patients may reflect the decoupling of functional brain networks underlying interoception, self-awareness, and appropriate emotional responses [37]. Besides, previous studies have shown that brain regions in the frontal-parietal cortex are central to the cognitive control process [38]. In particular, dmPFC activity typically indicates more top-down control over responses to emotional cues, while disruption of dmPFC function signifies less capacity of cognitive control during self-referential processing of sadness and may heighten the experiencing of negative affections so as to enhance vulnerability to subsequent depression [39]. Preliminary findings supported that youth who were better able to recruit prefrontal control regions exhibited greater resilience to depression following CM [40, 41].

While study the functions of the insula, bilateral insular subregions comprising anterior and posterior parts provided the most parsimonious solution [42, 43]. Specifically, the anterior insula, an important brain site for the perception of internal states and emotional regulation, is responsible for subjective emotions [17, 44], and integration of the homeostatic afferent signals from the posterior insula with emotional salience [45], as well as transmission of cognition processing information between the default mode network (DMN) and the cognitive control networks [46, 47]. Contrastingly, the posterior insula is proved to play a role in somatosensory stimuli responses with affective or motivational significance, including painful cutaneous stimuli, temperature sensation, thirst and hunger [48, 49]. Agree with this theory, the current study further demonstrated that the behavior significance of IFC networks were totally different between aIFC and pIFC. Overall, the CM experience and depressive symptoms had more extensive impact on the aIFC network than the pIFC network, suggests that the intrinsic connectivity of anterior insula network participates more in the neurobiological mechanism of depression pathogenesis, as an adverse consequence of maltreatment experiences. One potential explanation for these differential observations is that the dysregulation of mu opioid receptor (MOR) function may occur in particular in the anterior insular cortex [50, 51]. MOR is a G protein-coupled receptor that plays an essential role in reward and hedonic processes, and has been demonstrated to implicated in neuropsychiatric disorders especially depression [52]. Recent study in postmortem of depressed individual died by suicide has identified that enhanced endogenous opioidergic tone in the anterior insula may buffer negative affective states in MDD patients [53]. Previous literature confirmed that treatment-resistant depression patients had decreased regional homogeneity in the left insula [33], and had lower resting-state cerebral glucose uptake index in insula [54]. Besides, investigations of metabolic profiles at baseline found that metabolic disturbance in the insula was associated with nonresponse to cognitive-behavioral therapy and escitalopram [55]. These treatment-resistant properties of insula might explain why maltreated individuals were more likely to develop depression in adulthood, and were twice as likely to develop chronic or treatment-resistant depression than other risk factors [5].

In addition, our study investigated the associations between insula subregional networks and dimensional depressive symptoms. Notably, we found that among all the subtypes, childhood abuse had stronger association with anterior insula network in MDD patients than childhood neglect. Childhood abuse experiences were significantly correlated with extensive cortical-subcortical structures, including regions in frontal-parietal-occipital lobes, limbic system, and paralimbic area, as well as sensorimotor cortices. While childhood neglect experience were solely correlated with few regions, including bilateral dlPFC, dmPFC, SPL, MCC, PCC, middle temporal gyrus (MTG), and superior temporal gyrus (STG). Mechanistically, these findings suggested that individual differences in reported CM (abuse vs. neglect) had different impact on the specific neural circuits. These findings are in accordance with previous observations that childhood abuse represented a stronger association with depression than other forms of CM [56], and appear to specifically target brain regions (auditory, visual and somatosensory cortex) and circuits that convey and process the aversive experience [57]. Additionally, cortical thickness and volume in medial and lateral prefrontal and temporal lobes, as well as amygdala, Hip, and PHG, were reduced in children and adults with exposure to childhood abuse [58, 59], as such, regions like Hip, neocortex, and other structures may be most susceptible to childhood abuse [60]. Besides, accumulating evidence identified that these interconnecting regions have sensitive periods during development, and are most vulnerable to abuse exposure [57]. These symptom-specific correlations in the anterior and posterior insula networks connectivity may due to that childhood abuse involve severely environment threat and threats to one’s physical integrity or sexual violation [59].

In mediation analysis, we revealed that alterations in insula subregional connectivity could mediate the prospective association of childhood abuse and neglect with depressive symptoms in MDD patients. In terms of childhood abuse, the mediation brain pattern within aIFC overlapped most with the DMN and salience networks (SN). The Pcu, a major component of the DMN, is anatomically connected to PCC, ACC, and Tha [61] and have been proved to get involved in self-centered mental representation and first-person perspective-taking in function [62]. As mentioned above, dmPFC mainly participants in cognitive processes such as perspective-taking and social inferences [63], and help to regulate the degree of dorsal ACC and vmPFC activity underlying threat detection and response [8]. Previous studies also noted that the AI was actively connected with the ACC, which were two key composes of SN, jointly gave rise to feelings (insula) and motivations (ACC) that underlying all emotions, and played a critical role in self-awareness [64]. Additionally, other regions like Hip were implicated in memory and emotion processing and regulation, as well as emotional enhancement of memory representations [65]. In the present study, both AI and PI subregions showed altered connectivity with the dlPFC, which is the structural core in the executive control network (ECN) and responsible for manipulating the external information, processing evaluation, and determining the internal responses for action [66, 67]. Under the circumstances of childhood neglect, the PFC (including dlPFC, vmPFC, and vlPFC), within both aIFC and pIFC networks, play an important role in the mediating effect on depressive symptoms. Recently, Bojana and colleagues proposed a model that the dlPFC took charge of “update processing” that received the exogenous input, the vmPFC was responsible for “valuation”, while the dmPFC represented “cognitive” [68], as such, the coupling from dlPFC to vmPFC was suggested to be critical in reward evaluation and decision making [69]. Recently, literature also supposed that there was a triple network framework among DMN, SN, and ECN which characterized by shared alterations of functional connectivity architecture across psychiatric disorders, including depression [70–72]. Taken all of these evidence into consideration, the neural pathways underlying CM and increased susceptibility to depression may settle in the impairment of the triple network model among DMN, SN and ECN of the IFC networks. Specifically, the impairment of somatosensory/interoceptive information of the PI, then influence the self-regulation (including self-awareness, emotion regulation, and cognitive control) and decision-making, as well as corresponding behavior via AI.

Potential limitations of the present study include modest sample-size and sample-specific problems. All of the participants with MDD were integrated and investigated together. This limited the results in only depressive episode patients, while the neural pattern of CM experience subjects without depression, and the difference of neural circuit between depressive patients with and without CM are still unclear, which prevented us from digging into the interesting interactions between resilience and vulnerability. Moreover, our results may issue a hypothesis that exposure to different types of maltreatment could represent a clinically distinct subtype of MDD characterized by different symptom dimensions. Limited by the sample size, this hypothesis were not fully addressed. Future research could categorize into different types of maltreatment with different depressive symptoms to conduct this interesting investigation. In addition, although bilateral insula subregions comprising anterior and posterior parts provided the most parsimonious approach to study insula functions, the tripartite or more than three subregions provided more precise solution and has come into wide use [73, 74]. Later studies could improve from this aspect. Furthermore, future work could extend the framework to longitudinal settings to explore the dynamic influence of CM to depression and the dynamic mediating effect of altered brain networks.

## Conclusion

In conclusion, we reported preliminary evidence that, in MDD patients, the alterations of intrinsic connectivity of the bilateral insula networks were significantly correlated with CM experience. We also demonstrated that the RSFC of self-regulation and cognitive processing circuits could mediate the relationship between childhood abuse/neglect and depressive symptoms. These findings strongly suggest that CM leads to significant changes in the brain’s self-regulating and information processing circuitry, which will subsequently contribute to the occurrence of depression, and provide new insight into the understanding of pathological mechanism underlying CM-induced depressive disorder.

## Supplementary information

Supplemental Material
