# Supplementary material for: Insula network connectivity mediates the association between childhood maltreatment and depressive symptoms in major depressive disorder patients
Source: Transl Psychiatry. 2022 Mar 2;12:89. doi: 10.1038/s41398-022-01829-w (PMC8891292; doi:10.1038/s41398-022-01829-w)
Supplement: Supplementary file 1 — Supplemental Material [file 41398_2022_1829_MOESM1_ESM.docx]

**SUPPLEMENTARY MATERIAL**

**Title: Insula network connectivity mediates the association between childhood maltreatment and depressive symptoms in major depressive disorder patients**

This supplementary material includes:

Methods

Results

Table S1-S4

Figure S1-S2

**Supplementary Methods**

**MRI data acquisition**

Imaging was conducted on Siemens 3.0T scanner (Munich, Germany) with a homogeneous birdcage head coil at Henan Provincial Mental Hospital. All subjects were instructed to relax, maintain closed eyes and stay awake during scans. Earplugs were used to reduce noise and stabilizers were used to immobilize the heads. The T1 parameters were repetition time (TR) = 1900 ms, echo time (TE) = 2.48 ms, flip angle (FA) = 9°, acquisition matrix = 256 × 256, field of view (FOV) = 240 × 240 mm, thickness = 1.0 mm, gap = 0 mm, number of slices = 176, and number of excitations (NEX) = 1.0. The R-fMRI data were obtained over 8 min with a gradient-recalled echo-planar imaging (GRE-EPI) pulse sequence. The resting-state functional magnetic resonance image (R-fMRI) parameters included TR = 2000 ms, TE = 25 ms, FA = 90°, acquisition matrix = 64 × 64, FOV = 240 × 240 mm, thickness = 4.0 mm, gap = 0 mm, NEX = 1.0, and number of slices = 36. Additionally, routine axial T2-weighted images were obtained to exclude subjects with major white matter (WM) changes, cerebral infarction, or other lesions.

**Image preprocessing**

The fMRI images were preprocessed in the following steps: discarded the first ten volumes of the scanning session due to T1 equilibration effects. The remaining 230 volumes were corrected for slice timing, realigned, and were subsequently spatially normalized using the DARTEL toolbox[1] into Montreal Neurological Institute (MNI) space and smoothed with an 6-mm full-width half-maximum kernel. To further reduce the effects of confounding factors, friston 24 motion parameters, the global mean signal, white matter (WM) signal, and cerebrospinal fluid (CSF) signal were removed from the data via linear regression. We also calculated the framewise displacement (FD), which reflects the mismatch of volume-to-volume head position[2, 3]. There was no significant difference in the FD among the groups (p > 0.05), and the mean FD was also applied as a covariate in the imaging analyses. Moreover, a bandpass filter was applied to maintain low-frequency fluctuations within a frequency range of 0.01-0.08 Hz.

**Structural image analysis**

An optimized voxel-based morphometry (VBM) analysis was conducted using SPM8 to calculate the GM volume in all subjects. The T1-weighted images were segmented into GM, white matter, and CSF, and subsequently, the segmented GM was normalized and smoothed with a 6-mm Full Width Half Maximum (FWHM) Gaussian kernel. To avoid the bias of the functional connectivity strength derived from anatomical variations, gray matter (GM) volume was considered as an important covariate in the functional connectivity analysis[4]. The GM volume was regressed out as a covariate to control for the effects on the functional connectivity strength.

**Voxel-wise based insula functional connectivity construction**

We created spherical ROIs of 6 mm radius for bilateral insula, included bilateral AI ([–30, 20, 6] and [30, 26, 4]) and bilateral PI ([–40, 4, –4] and [42, –6, –8]), to obtain the anterior and posterior insula functional connectivity (IFC) map for each participant. The averaged time series of each insula region was used as the seed time series. Then, a Pearson cross-correlation was used to calculate the correlation coefficients (CC) between the seed regions and all brain voxels as the strength of the functional connectivity. Subsequently, Fisher's Z-transformation was applied to improve the correlation coefficient values to approach a normal distribution [m = 0.5 ln(1+CC)/(1−CC)][5]. These voxel-wised whole-brain values were resampled to 3-mm isotropic voxels and smoothed with a 6-mm FWHM Gaussian kernel. As a result, IFC map for each individual subject was obtained.

**Mediation analysis**

The classic mediation model was selected and then Sobel test was used to confirm the significance of the mediator if the weighted coefficient (a or b) was not significance[6, 7]. Three steps regression models were constructed, as shown below:


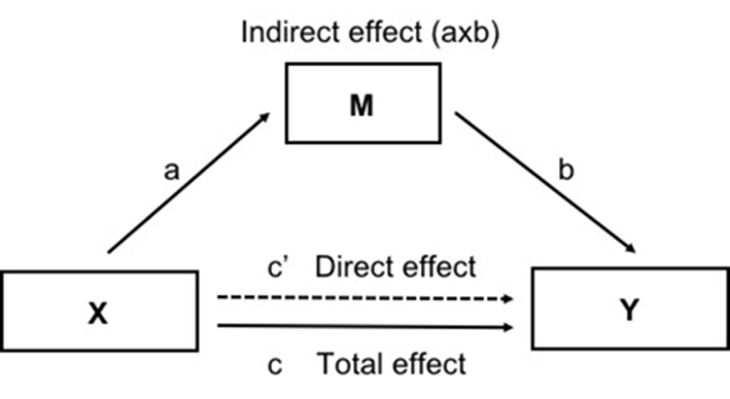


Y = *c*X + *e*1 (1)

M = *a*X + *e*2 (2)

Y = *c’*X + *b*M + *e*3 or $z=\frac{ab}{\sqrt{{(b}^{2}\mathrm{SE}_{a}^{2})-({a^{2}\mathrm{SE}}_{b}^{2})}}$ (3)

X is the dependent variable, Y is the independent variable, M is the mediator, a is the regression coefficient for the relationship between X and M, b is the regression coefficient for the relationship between M and Y, c is the regression coefficient for the relationship between X on Y, z and c’ represent the effect of X on Y while controlling for the indirect effect. SEa is the standard error of the relationship between X and M, and SEb is the standard error of the relationship between M and Y.

In this analysis, four conditions for establishing mediation are: 1) c must be significant; 2) a and b are significant; 3) c’ < c (in absolute value, partial mediation) or c’ is insignificant (full mediation); 4) if a or b is insignificant, the Sobel test Z must be significant. If mediation was presented, we used ratio indirect to present the strength of mediation ((a*b)/c). In the process of SPSS syntax, an indirect effect was considered significant if 95% bias-corrected confidence intervals (CI) from bootstrapped analyses (10,000 resamples) did not contain zero.

**Supplementary Results**

**Intrinsic connectivity patterns of bilateral anterior and posterior IFC networks in MDD and HC subjects**

Each IFC network is composed of both positive and negative network. Specifically, as shown in Fig. S1A, for the HCs, the left aIns showed significantly positive connectivity with the bilateral dorsal lateral prefrontal cortex (dlPFC), inferior frontal gyrus (IFG), supplementary motor area (SMA), anterior cingulate cortex (ACC), inferior parietal cortex (IPC), caudate, putamen, thalamus, and hippocampus. Significantly negative connectivity was evident as related to the bilateral ventral medial prefrontal cortex (vmPFC), posterior cingulate cortex (PCC), precuneus, and cerebellum. The positive right aIns network consisted of the bilateral, IFG, SMA, dorsal ACC (dACC), middle cingulate cortex (MCC), IPC, caudate, putamen, thalamus, and hippocampus. The negative right aIns network included the bilateral PCC, precuneus and the cerebellum. The positive left pIns network consisted of the bilateral IFG, SMA, IPC and subcortical regions, including the caudate, putamen, thalamus, and hippocampus. The negative left pIns network included the bilateral superior frontal gyrus (SFG), right dlPFC, PCC, precuneus and the cerebellum. The positive right pIns network was seen in the bilateral IFG, SMA, thalamus and hippocampus. The negative left pIns network included the bilateral SFG, dmPFC, PCC, precuneus and the cerebellum. For MDD patients, the patterns of the IFC networks were presented in Fig. S1B.

**Main effects of depressive symptoms on IFC networks in MDD patients**

Voxel-wised multivariate linear regression analysis identified the neural effects of HAMD-17 total and subscales scores on the bipartite IFC networks, as shown in **Fig. S2**. On the whole, the main effects of HAMD-17 subscales scores on the IFC networks were broader than the main effects of HAMD-17 total scores. Secondly, the main effects of HAMD-17 scores on the aIFC network **(Fig. S2A)** were borader than pIFC network **(Fig. S2B)**. Thirdly, HAMD-anxiety scores had a broader influence on the aIFC network and the significant brain regions were mainly located in frontal-occipital-limbic system **(Fig. S2A(b))**. Meanwhile, HAMD-cognition had extensive impact on the pIFC network and the significant brain regions were mainly observed in frontal-parietal areas **(Fig. S2B(e)).**

**References**

1. Ashburner, J., *A fast diffeomorphic image registration algorithm.* Neuroimage, 2007. **38**(1): p. 95-113.

2. Power, J.D., et al., *Steps toward optimizing motion artifact removal in functional connectivity MRI; a reply to Carp.* Neuroimage, 2013. **76**: p. 439-41.

3. Power, J.D., et al., *Spurious but systematic correlations in functional connectivity MRI networks arise from subject motion.* Neuroimage, 2012. **59**(3): p. 2142-54.

4. Xie, C., et al., *Joint effects of gray matter atrophy and altered functional connectivity on cognitive deficits in amnestic mild cognitive impairment patients.* Psychol Med, 2015. **45**(9): p. 1799-810.

5. Xie, C., et al., *Abnormal insula functional network is associated with episodic memory decline in amnestic mild cognitive impairment.* Neuroimage, 2012. **63**(1): p. 320-7.

6. Baron, R.M. and D.A. Kenny, *The moderator-mediator variable distinction in social psychological research: conceptual, strategic, and statistical considerations.* J Pers Soc Psychol, 1986. **51**(6): p. 1173-82.

7. Sobel, M.E., *Asymptotic Confidence Intervals For Indirect Effects In Structural Equation Models.* Sociological Methodology, 1982: p. 290-312.

**Table S1. Brain regions with altered insula functional connectivity (IFC) in the MDD patients compared with HC subjects.**

| Brain region | BA | Cluster size  (mm^3^) | MNI Coordinate  (RAI) | | | Peak *Z* score |
| --- | --- | --- | --- | --- | --- | --- |
|  |  |  | X | Y | Z |  |
| **anterior IFC Network** | | | | | | |
| **MDD<HC** | | | | | | |
| RdmPFC | 10 | 5940 | 6 | 69 | 9 | -3.17 |
| **MDD>HC** | | | | | | |
| RMFG | 6 | 3915 | 33 | -3 | 51 | 2.88 |
| **Posterior IFC Network** | | | | | | |
| **MDD<HC** | | | | | | |
| bdmPFC | 10 | 10098 | 15 | 45 | -3 | -3.12 |
| **MDD>HC** | | | | | | |
| bMFG | 6 | 4185 | -3 | -21 | 54 | 3.15 |

**Abbreviation:** IFC, insula functional connectivity; HC, healthy control; MDD, major depressive disorder; BA, brodmann area; RdmPFC, right dorsal medial prefrontal cortex; bSMA, bilateral supplementary motor area; LPut, left putamen; RPut/LN, right putamen/lentiform nucleus; LFFA, left fusiform area; RCau/Put, right caudate nucleus/putamen; LvmPFC, left ventral medial prefrontal cortex; LLN, left lentiform nucleus.

**Table S2. Neural effects of CTQ total and sub-factors overlapped with HAMD-17 total and sub-factors on anterior insula functional connectivity network in MDD patients.**

| Brain regions | BA | Cluster size (mm^3^) | MNI coordinates (RAI) | | |
| --- | --- | --- | --- | --- | --- |
|  |  |  | x | y | z |
| **Overlapping effects of CTQ and HAMD-17** | | | | | |
| RSMA | 6 | 1728 | 9 | -15 | 72 |
| LCal | 18 | 1323 | -15 | -72 | 12 |
| LSTG | 13 | 1161 | -48 | -42 | 15 |
| RPcu | 7 | 1161 | 6 | -69 | 54 |
| LvlPFC | 11 | 729 | -39 | 36 | -12 |
| RPCG | 4/3 | 702 | 33 | -30 | 66 |
| LIFG | 10 | 675 | -39 | 45 | 6 |
| RPCC | 29 | 351 | 9 | -48 | 15 |
| RvmPFC | 11 | 216 | 6 | 54 | -12 |
| **Overlapping effects of CTQ and HAMD-Weight** | | | | | |
| LPcu | 7 | 972 | 0 | -57 | 51 |
| RdlPFC | 10 | 432 | 24 | 63 | 18 |
| **Overlapping effects of CTQ and HAMD-Sleep** | | | | | |
| RSMA | 6 | 945 | 3 | -9 | 72 |
| LMCC | 24 | 486 | -12 | 0 | 39 |
| **Overlapping effects of CTQ and HAMD-Anxiety** | | | | | |
| bPCC | 30 | 5697 | 9 | -39 | 0 |
| RdlPFC | 45 | 3024 | 60 | 21 | 21 |
| RLG | 18 | 2808 | 21 | -90 | -9 |
| ROFC | 11 | 729 | 9 | 51 | -15 |
| RdmPFC | 9 | 432 | 12 | 54 | 36 |
| LvmPFC | 32 | 297 | -6 | 45 | -9 |
| **Overlapping effects of CTQ and HAMD-Cognition** | | | | | |
| LvlPFC | 47 | 6264 | -45 | 33 | -15 |
| RSPL | 6 | 2646 | 27 | -24 | 63 |
| LIPL | 3/4 | 1512 | -24 | -33 | 54 |
| RMCC | NA | 891 | 12 | -6 | 36 |
| RdmPFC | 10 | 621 | 3 | 57 | 6 |
| RSMG | 9 | 567 | 6 | 54 | 30 |
| **Overlapping effects of CTQ and HAMD-Retardation** | | | | | |
| RCal | 23 | 1971 | 6 | -75 | 9 |
| RMCC | NA | 1593 | 12 | -9 | 36 |
| LdlPFC | 10 | 1080 | -39 | 42 | 3 |
| LPCL | 6/3 | 648 | -15 | -30 | 60 |
| **Overlapping effects of CTQ-SA and HAMD-17** | | | | | |
| bACC | 32 | 9747 | 6 | 30 | -6 |
| LdmPFC | 10 | 5724 | -18 | 57 | 16 |
| bSMA | 6 | 5616 | -3 | -12 | 57 |
| Rput | NA | 4563 | 18 | 9 | -9 |
| RPcu | 7 | 3267 | 6 | -72 | 57 |
| LvlPFC | 47 | 1269 | -42 | 30 | -12 |
| **Overlapping effects of CTQ-SA and HAMD-Weight** | | | | | |
| RPcu | 7 | 2106 | 6 | -66 | 54 |
| RdlPFC | 10 | 1782 | 27 | 57 | 3 |
| LdlPFC | 10 | 1782 | -18 | 54 | 9 |
| RMCC | NA | 189 | 9 | -18 | 30 |
| **Overlapping effects of CTQ-SA and HAMD-Sleep** | | | | | |
| LdlPFC | 6 | 2133 | -21 | -6 | 57 |
| LMCC | NA | 432 | -12 | 0 | 39 |
| **Overlapping effects of CTQ-SA and HAMD-Anxiety** | | | | | |
| bPCC | NA | 7074 | 3 | -45 | 28 |
| RvlPFC | 10 | 3780 | 39 | 57 | 0 |
| RdlPFC | 44 | 2619 | 60 | 6 | 21 |
| RdmPFC | 32 | 2322 | 15 | 45 | 0 |
| LACC | 32 | 1269 | -9 | 36 | -3 |
| LCau | NA | 486 | -12 | 12 | 12 |
| RTha | NA | 324 | 6 | -24 | 12 |
| **Overlapping effects of CTQ-SA and HAMD-Cognition** | | | | | |
| LIns | 38 | 14067 | -27 | 18 | -18 |
| bSMA | 6 | 9936 | 9 | 6 | 51 |
| LvmPFC | 10 | 1836 | -9 | 54 | -15 |
| RdmPFC | 10 | 1701 | 15 | 48 | 0 |
| bACC | 33 | 1269 | 0 | 18 | 24 |
| RvlPFC | 11 | 1161 | 45 | 33 | -18 |
| RMCC | 24 | 837 | 6 | -9 | 36 |
| **Overlapping effects of CTQ-SA and HAMD-Retardation** | | | | | |
| LOFC | 47 | 8910 | -33 | 30 | -18 |
| RdlPFC | 19 | 6777 | 26 | 53 | 9 |
| RTP | 38 | 3483 | 51 | 18 | -18 |
| RMCC | NA | 3024 | 6 | -18 | 33 |
| LdlPFC | 10 | 3267 | -29 | 49 | 13 |
| RCau | NA | 1782 | 12 | 18 | 10 |
| LCau | NA | 729 | -15 | 21 | 3 |
| **Overlapping effects of CTQ-PA and HAMD-17** | | | | | |
| bPcu | 7 | 2403 | -3 | -54 | 54 |
| LdlPFC | 10 | 378 | -30 | 36 | 15 |
| LHip | 34 | 216 | -21 | -9 | -21 |
| LPHG | 35 | 162 | -21 | -6 | -30 |
| **Overlapping effects of CTQ-PA and HAMD-Weight** | | | | | |
| RPcu | 7 | 1107 | 9 | -69 | 57 |
| RTha | 23 | 486 | 3 | -21 | 21 |
| **Overlapping effects of CTQ-PA and HAMD-Sleep** | | | | | |
| LdlPFC | 8 | 594 | -18 | 21 | 48 |
| **Overlapping effects of CTQ-PA and HAMD-Anxiety** | | | | | |
| bTha | NA | 1134 | 3 | -21 | 3 |
| LPCC | 23 | 1107 | -9 | -48 | 18 |
| RPcu | 7 | 918 | 9 | -69 | 57 |
| LPHG | 34/35 | 810 | -21 | -6 | -33 |
| LdlPFC | 8 | 540 | 9 | 33 | 57 |
| LMOG | 17 | 405 | -21 | -93 | 0 |
| **Overlapping effects of CTQ-PA and HAMD-Cognition** | | | | | |
| RPcu | 7 | 3834 | 18 | -48 | 45 |
| LdlPFC | 13 | 1674 | -42 | 21 | 15 |
| RMCC | NA | 918 | 12 | -6 | 36 |
| RdmPFC | 32 | 783 | 9 | 6 | 51 |
| LdmPFC | 9 | 405 | -12 | 30 | 42 |
| **Overlapping effects of CTQ-PA and HAMD-Retardation** | | | | | |
| bCun | 7 | 2754 | 6 | -75 | 27 |
| RMCC | NA | 1485 | 15 | -15 | 36 |
| **Overlapping effects of CTQ-PN and HAMD-17** | | | | | |
| RdlPFC | 10 | 756 | 39 | 33 | 3 |
| **Overlapping effects of CTQ-PN and HAMD-Weight** | | | | | |
| RdlPFC | 10 | 1350 | 30 | 60 | 0 |
| **Overlapping effects of CTQ-PN and HAMD-Anxiety** | | | | | |
| RdlPFC | 10 | 1917 | 37 | 54 | 12 |
| **Overlapping effects of CTQ-PN and HAMD-Anxiety** | | | | | |
| RvlPFC | 47 | 675 | 42 | 36 | 3 |
| ROFC | 47/11 | 189 | 45 | 33 | -15 |
| **Overlapping effects of CTQ-PN and HAMD-Retardation** | | | | | |
| RdlPFC | 47 | 2214 | 40 | 30 | 8 |
| **Overlapping effects of CTQ-EA and HAMD-17** | | | | | |
| LdlPFC | 10 | 2322 | -18 | 51 | 12 |
| RSMA | 6 | 1404 | 9 | -15 | 72 |
| LCal | 30 | 1296 | -15 | -72 | 12 |
| RPcu | 7 | 1242 | 6 | -72 | 51 |
| RIns | 13 | 729 | 27 | 21 | -9 |
| LvlPFC | 11 | 702 | -39 | 39 | -9 |
| **Overlapping effects of CTQ-EA and HAMD-Weight** | | | | | |
| RPcu | 7 | 1890 | 5 | -72 | 54 |
| LdlPFC | 10 | 1566 | -21 | 54 | 12 |
| **Overlapping effects of CTQ-EA and HAMD-Sleep** | | | | | |
| RSMA | 6 | 702 | 3 | -9 | 72 |
| **Overlapping effects of CTQ-EA and HAMD-Anxiety** | | | | | |
| bPCC | 30 | 8127 | 6 | -50 | 20 |
| RdlPFC | 9 | 3699 | 57 | 21 | 18 |
| RAG | NA | 2970 | 51 | -57 | 27 |
| RLG | 18 | 2403 | 18 | -91 | -6 |
| RvmPFC | 11 | 1512 | 9 | 51 | -15 |
| **Overlapping effects of CTQ-EA and HAMD-Cognition** | | | | | |
| LOFC/vlPFC | 47 | 2133 | -45 | 33 | -15 |
| RSPL | 4 | 2079 | 21 | -33 | 66 |
| LdlPFC | 10 | 1863 | -21 | 57 | 12 |
| ROFC | 11 | 1674 | 36 | 39 | -18 |
| RMCC | 33 | 540 | 6 | 12 | 30 |
| RdmPFC | 10 | 351 | 3 | 60 | 3 |
| **Overlapping effects of CTQ-EA and HAMD-Retardation** | | | | | |
| LdlPFC | 10 | 2565 | -39 | 42 | 3 |
| LCun | NA | 2106 | -8 | -75 | 22 |
| RPut | 13 | 1242 | 27 | 18 | -6 |
| RdlPFC | 10 | 405 | 33 | 48 | 0 |
| LIns | 13 | 324 | -42 | 15 | 6 |
| RCau | NA | 243 | 12 | 15 | 12 |
| **Overlapping effects of CTQ-EN and HAMD-17** | | | | | |
| RdlPFC | 10 | 567 | 19 | 62 | 22 |
| RvmPFC | 11 | 297 | 6 | 51 | -12 |
| **Overlapping effects of CTQ-EN and HAMD-Weight** | | | | | |
| RdlPFC | 10 | 189 | 21 | 63 | 21 |
| **Overlapping effects of CTQ-EN and HAMD-Anxiety** | | | | | |
| RPCC | NA | 594 | 9 | -42 | 10 |
| RvmPFC | 11 | 432 | 9 | 51 | -15 |
| LvmPFC | 32 | 324 | -9 | 42 | -12 |
| **Overlapping effects of CTQ-EN and HAMD-Cognition** | | | | | |
| RdmPFC | 10 | 945 | 9 | 57 | 3 |
| **Overlapping effects of CTQ-EN and HAMD-Retardation** | | | | | |
| LIPL | 4 | 648 | -27 | -27 | 57 |
| LSPL | 6 | 729 | -15 | -30 | 60 |
| RdlPFC | 10 | 378 | 18 | 63 | 21 |

**Abbreviation:** MDD, Major Depressive Disorder; HAMD-17, 17-items Hamilton Depression Scale; HAMD-Anxiety, HAMD-17 Anxiety/somatization subscale; HAMD-Weight, HAMD-17 Weight loss subscale; HAMD-Cognition, HAMD-17 Cognitive disturbance subscale; HAMD-Retardation, HAMD-17 Retardation subscale; HAMD-Sleep, HAMD-17 Sleep disruption subscale; CTQ, Childhood Trauma Questionnaire; CTQ-PA, CTQ Physical Abuse subscale; CTQ-EA, CTQ Emotional Abuse subscale; CTQ-SA, CTQ Sexual Abuse subscale; CTQ-PN, CTQ Physical Neglect subscale; CTQ-EN, CTQ Emotional Neglect subscale; RSMA, right supplementary motor area; LCal, left calcarine gyrus; LSTG, left superior temporal gyrus; RPcu, right precuneus; LvlPFC, left ventral lateral prefrontal cortex; RPCG, right Right postcentral gyrus; LIFG, left inferior frontal gyrus; RPCC, right posterior cingulate cortex; RvmPFC, right ventral medial prefrontal cortex; LPcu, left precuneus; RdlPFC, right dorsal lateral prefrontal cortex; LMCC, left middle cingulate cortex; bPCC, bilateral posterior cingulate cortex; RLG, right lingual gyrus; ROFC, right orbitofrontal cortex; RdmPFC, right dorsal medial prefrontal cortex; LvmPFC, left ventral medial prefrontal cortex; RSPL, right superior parietal lobe; LIPL, left inferior parietal lobe; RMCC, right middle cingulate cortex; RSMG, right supramarginal gyrus; RCal, right calcarine gyrus; LdlPFC, left dorsal lateral prefrontal cortex; LPCL, Left Paracentral Lobule; bACC, bilateral anterior cingulate cortex; LdmPFC, left dorsal medial prefrontal cortex; bSMA, bilateral supplementary motor area; RPut, right putamen; RvlPFC, right ventral lateral prefrontal cortex; LACC, left anterior cingulate cortex; LCau, left caudate nucleus; RTha, right thalamus; LIns, left insula; LOFC, left orbitofrontal cortex; RTP, right temporal pole; RCau, right caudate nucleus; bPcu, bilateral precuneus; LHip, left hippocampus; LPHG, left parahippocampus; bTha, bilateral thalamus; LPCC, left posterior cingulate cortex; LMOG, left middle occipital lobe; bCun, bilateral cuneus; RIns, right insula; RAG, right angular gyrus; RLG, right lingual gyrus; LCun, left cuneus; LSPL, left superior parietal lobe.

**Table S3. Neural effects of CTQ total and sub-factors overlapped with HAMD-17 total and sub-factors on posterior insula functional connectivity network in MDD patients.**

| Brain regions | BA | Cluster size (mm^3^) | MNI coordinates (RAI) | | |
| --- | --- | --- | --- | --- | --- |
|  |  |  | x | y | z |
| **Overlapping effects of CTQ and HAMD-17** | | | | | |
| RCun | 19 | 864 | 15 | -78 | 39 |
| RMTG | 39 | 459 | 51 | -57 | 0 |
| **Overlapping effects of CTQ and HAMD-Sleep** | | | | | |
| RPcu | 19 | 1782 | 15 | -78 | 39 |
| **Overlapping effects of CTQ and HAMD-Cognition** | | | | | |
| RFFA | 37 | 378 | 39 | -63 | -18 |
| **Overlapping effects of CTQ and HAMD-Retardation** | | | | | |
| RIPL | 7 | 513 | 36 | -45 | 42 |
| **Overlapping effects of CTQ-SA and HAMD-17** | | | | | |
| LPcu | 7 | 972 | -12 | -75 | 30 |
| **Overlapping effects of CTQ-SA and HAMD-Weight** | | | | | |
| RdlPFC | 9 | 972 | 36 | 15 | 33 |
| **Overlapping effects of CTQ-SA and HAMD-Sleep** | | | | | |
| RITG | 21 | 675 | 63 | -36 | -27 |
| RSOG | 7 | 405 | 24 | -78 | 36 |
| **Overlapping effects of CTQ-SA and HAMD-Anxiety** | | | | | |
| LvlPFC | 47 | 2835 | 51 | 39 | 0 |
| RdlPFC | 45 | 2079 | 60 | 21 | 18 |
| **Overlapping effects of CTQ-SA and HAMD-Cognition** | | | | | |
| LPcu | 4 | 1701 | -15 | -63 | 69 |
| RIPL | 3/4 | 1269 | 18 | -39 | 69 |
| **Overlapping effects of CTQ-SA and HAMD-Retardation** | | | | | |
| LdlPFC | 45 | 7857 | -51 | 6 | 25 |
| RdlPFC | 46 | 7290 | 48 | 18 | 31 |
| LvlPFC | 10 | 5076 | -36 | 36 | -12 |
| bdmPFC | 9 | 4455 | -3 | 48 | 27 |
| LPut | NA | 2079 | -18 | 9 | 0 |
| RIns | NA | 567 | 27 | 21 | -9 |
| **Overlapping effects of CTQ-PA and HAMD-17** | | | | | |
| RPCC | 29 | 999 | -15 | 36 | 0 |
| **Overlapping effects of CTQ-PA and HAMD-Sleep** | | | | | |
| RHip/PHG | 27 | 2619 | 21 | -45 | -6 |
| LHip/PHG | 27 | 918 | -18 | -36 | -6 |
| **Overlapping effects of CTQ-PA and HAMD-Anxiety** | | | | | |
| RvlPFC | 45 | 2538 | 57 | 36 | 0 |
| LHip | 27/30 | 864 | -18 | -33 | -3 |
| **Overlapping effects of CTQ-PA and HAMD-Retardation** | | | | | |
| RdlPFC | 45 | 2133 | 43 | 27 | 9 |
| RvlPFC | 10 | 1539 | 54 | 33 | 3 |
| **Overlapping effects of CTQ-PN and HAMD-17** | | | | | |
| RPcu | 7 | 1674 | 8 | -74 | 47 |
| **Overlapping effects of CTQ-PN and HAMD-Sleep** | | | | | |
| RPcu | 7 | 4077 | 30 | -72 | 33 |
| LHip/PHG | 30 | 567 | -21 | -36 | -9 |
| **Overlapping effects of CTQ-PN and HAMD-Anxiety** | | | | | |
| RdlPFC | 10 | 783 | 42 | 48 | 3 |
| LHip | 30 | 270 | -21 | -36 | -3 |
| **Overlapping effects of CTQ-PN and HAMD-Cognition** | | | | | |
| LdlPFC | 9 | 918 | -24 | 42 | 24 |
| **Overlapping effects of CTQ-PN and HAMD-Retardation** | | | | | |
| bdmPFC | 9 | 1080 | -3 | 48 | 27 |
| **Overlapping effects of CTQ-EA and HAMD-Anxiety** | | | | | |
| RdlPFC | 10 | 1917 | 33 | 42 | 9 |
| **Overlapping effects of CTQ-EA and HAMD-Retardation** | | | | | |
| LdlPFC | 10/46 | 2403 | -33 | 39 | -6 |
| LdmPFC | 32/6 | 1269 | -6 | 39 | 33 |
| RIns | 13 | 324 | 27 | 21 | -9 |
| **Overlapping effects of CTQ-EN and HAMD-17** | | | | | |
| RPcu | 7 | 999 | 27 | -69 | 36 |
| **Overlapping effects of CTQ-EN and HAMD-Sleep** | | | | | |
| RPcu | 7 | 2403 | 16 | -76 | 46 |
| LHip/PHG | NA | 594 | -18 | -36 | -6 |
| **Overlapping effects of CTQ-EN and HAMD-Anxiety** | | | | | |
| LHip | 35 | 513 | -24 | -36 | -6 |
| **Overlapping effects of CTQ-EN and HAMD-Cognition** | | | | | |
| LIPL | 3 | 2133 | -25 | -27 | 63 |
| LMCC | NA | 1053 | -6 | -18 | 33 |
| RvlPFC | 22 | 675 | -51 | 3 | -3 |
| LIns | NA | 378 | -36 | -9 | 9 |
| **Overlapping effects of CTQ-EN and HAMD-Retardation** | | | | | |
| LdlPFC | 9 | 2619 | -54 | 4 | 26 |
| LIPL | 40 | 2268 | -36 | -50 | 43 |

**Abbreviation:** RCun, right cuneus; RMTG, right middle temporal gyrus; RPcu, right precuneus; RFFA, right fusiform area; RIPL, right inferior parietal lobe; LPcu, left precuneus; RdlPFC, right dorsal lateral prefrontal cortex; RITG, right inferior temporal gyrus; RSOG, right superior occipital gyrus; LvlPFC, left ventral lateral prefrontal cortex; LdlPFC, left dorsal lateral prefrontal cortex; bdmPFC, bilateral dorsal medial prefrontal cortex; LPut, left putamen; RIns, right insula; RPCC, right posterior cingulate cortex; RHip/PHG, right hippocampus/parahippocampus; LHip/PHG, left hippocampus/parahippocampus; RvlPFC, right ventral lateral prefrontal cortex; LHip, left hippocampus; LdmPFC, left dorsal medial prefrontal cortex; LIPL, left inferior parietal lobe; LMCC, left middle cingulate cortex; LIns, left insula.

**Table S4. Brain regions within the aIFC and pIFC networks that could mediate the association between childhood abuse and neglect and depressive symptoms.**

| **X** | **Y** | **Brain regions** | **Indirect effect** | **Bootstrap**  **95% CI of a*b** | | **Boot SE** |
| --- | --- | --- | --- | --- | --- | --- |
| **anterior IFC Network** | | | | | | |
| CTQ | Hamd17 | RPcu | 0.0412 | 0.0058 | 0.1154 | 0.0261 |
| CTQ | Hamd17-W | LPcu | 0.0043 | 0.0009 | 0.0106 | 0.0023 |
| CTQ | Hamd17-A | LPCC | -0.0246 | -0.0471 | -0.0103 | -0.0103 |
| CTQ | Hamd17-A | RdlPFC | -0.0268 | -0.0535 | -0.0106 | 0.0107 |
| CTQ | Hamd17-C | LvlPFC | 0.0108 | 0.0045 | 0.0205 | 0.0040 |
| CTQ | Hamd17-C | RMCC | 0.0062 | 0.0004 | 0.0172 | 0.0041 |
| CTQ | Hamd17-RD | LdlPFC | 0.0185 | 0.0042 | 0.0445 | 0.0097 |
| CTQ-SA | Hamd17 | RACC | 0.3917 | 0.164 | 0.6715 | 0.1291 |
| CTQ-SA | Hamd17 | LdmPFC | 0.3593 | 0.0746 | 0.8881 | 0.2077 |
| CTQ-SA | Hamd17 | RCau | 0.3095 | 0.102 | 0.6099 | 0.1258 |
| CTQ-SA | Hamd17 | LCau | 0.2579 | 0.0502 | 0.6033 | 0.1351 |
| CTQ-SA | Hamd17 | RPcu | 0.2973 | 0.0895 | 0.6173 | 0.1306 |
| CTQ-SA | Hamd17 | LvlPFC | 0.2413 | 0.0705 | 0.5022 | 0.1076 |
| CTQ-SA | Hamd17-W | RPcu | 0.0323 | 0.0106 | 0.0667 | 0.0138 |
| CTQ-SA | Hamd17-W | RdlPFC | 0.0397 | 0.0103 | 0.0998 | 0.0219 |
| CTQ-SA | Hamd17-W | LdlPFC | 0.0409 | 0.012 | 0.1048 | 0.0239 |
| CTQ-SA | Hamd17-W | RMCC | -0.0286 | -0.07 | -0.0056 | 0.0163 |
| CTQ-SA | Hamd17-S | LdlPFC | 0.2039 | 0.082 | 0.42 | 0.0858 |
| CTQ-SA | Hamd17-S | LMCC | 0.1159 | 0.0255 | 0.2772 | 0.0639 |
| CTQ-SA | Hamd17-A | bPCC | -0.1887 | -0.3884 | -0.0746 | 0.0780 |
| CTQ-SA | Hamd17-A | RvlPFC | -0.1859 | -0.3785 | -0.0747 | 0.0737 |
| CTQ-SA | Hamd17-A | LACC | 0.1592 | 0.0673 | 0.3137 | 0.0608 |
| CTQ-SA | Hamd17-A | LCau | 0.1461 | 0.0469 | 0.3411 | 0.0730 |
| CTQ-SA | Hamd17-A | RTha | -0.0817 | -0.1794 | -0.0217 | 0.0387 |
| CTQ-SA | Hamd17-C | LvmPFC | 0.0677 | 0.029 | 0.1249 | 0.0243 |
| CTQ-SA | Hamd17-C | RdmPFC | 0.0511 | 0.0208 | 0.1044 | 0.0206 |
| CTQ-SA | Hamd17-C | bACC | -0.0427 | -0.0852 | -0.0112 | 0.0184 |
| CTQ-SA | Hamd17-RD | LdlPFC | 0.4267 | 0.227 | 0.6888 | 0.1150 |
| CTQ-SA | Hamd17-RD | RCau | 0.3231 | 0.1669 | 0.5465 | 0.0931 |
| CTQ-SA | Hamd17-RD | RMCC | 0.2345 | 0.101 | 0.4143 | 0.0783 |
| CTQ-SA | Hamd17-RD | LCau | 0.1690 | 0.0582 | 0.3848 | 0.0748 |
| CTQ-EA | Hamd17 | LdlPFC | 0.1900 | 0.0719 | 0.3804 | 0.0759 |
| CTQ-EA | Hamd17 | RPcu | 0.1494 | 0.0306 | 0.3887 | 0.0859 |
| CTQ-EA | Hamd17-W | RPcu | 0.0210 | 0.008 | 0.0454 | 0.0088 |
| CTQ-EA | Hamd17-W | LdlPFC | 0.0173 | 0.0062 | 0.0368 | 0.0074 |
| CTQ-EA | Hamd17-A | RPCC | -0.1012 | -0.2056 | -0.038 | 0.0407 |
| CTQ-EA | Hamd17-A | RdlPFC | -0.1121 | -0.2192 | -0.0428 | 0.0443 |
| CTQ-EA | Hamd17-A | RvmPFC | -0.0666 | -0.144 | -0.0229 | 0.0292 |
| CTQ-EA | Hamd17-C | LOFC | 0.0297 | 0.0123 | 0.058 | 0.0110 |
| CTQ-EA | Hamd17-C | ROFC | 0.0301 | 0.0118 | 0.0614 | 0.0120 |
| CTQ-EA | Hamd17-C | RMCC | -0.0299 | -0.0583 | -0.01 | 0.0121 |
| CTQ-EA | Hamd17-RD | LdlPFC | 0.1212 | 0.0534 | 0.2245 | 0.0427 |
| CTQ-EA | Hamd17-RD | RPut | 0.0423 | 0.0026 | 0.1222 | 0.0295 |
| CTQ-EA | Hamd17-RD | RdlPFC | 0.0714 | 0.0246 | 0.152 | 0.0309 |
| CTQ-EA | Hamd17-RD | LIns | 0.0762 | 0.016 | 0.1885 | 0.0419 |
| CTQ-EA | Hamd17-RD | RCau | 0.0603 | 0.0136 | 0.1621 | 0.0343 |
| CTQ-PA | Hamd17 | bPcu | 0.3110 | 0.0869 | 0.6659 | 0.1429 |
| CTQ-PA | Hamd17 | LdlPFC | 0.2189 | 0.0303 | 0.5343 | 0.1264 |
| CTQ-PA | Hamd17 | LHip | 0.1658 | 0.0368 | 0.4063 | 0.0902 |
| CTQ-PA | Hamd17 | LPHG | 0.1370 | 0.0083 | 0.3726 | 0.0866 |
| CTQ-PA | Hamd17-W | RPcu | 0.0309 | 0.008 | 0.0713 | 0.0152 |
| CTQ-PA | Hamd17-S | LdlPFC | -0.1295 | -0.2366 | -0.0453 | 0.0476 |
| CTQ-PA | Hamd17-A | bTha | -0.1920 | -0.3206 | -0.0828 | 0.0615 |
| CTQ-PA | Hamd17-A | LPCC | -0.1626 | -0.2866 | -0.0707 | 0.0536 |
| CTQ-PA | Hamd17-A | RPcu | 0.0996 | 0.0181 | 0.24 | 0.0553 |
| CTQ-PA | Hamd17-A | LPHG | 0.1425 | 0.0621 | 0.2864 | 0.0550 |
| CTQ-PA | Hamd17-A | LdlPFC | 0.0912 | 0.0216 | 0.1887 | 0.0422 |
| CTQ-PA | Hamd17-C | RPcu | 0.0510 | 0.0217 | 0.0892 | 0.0169 |
| CTQ-PA | Hamd17-C | LdlPFC | 0.0309 | 0.0045 | 0.0756 | 0.0171 |
| CTQ-PA | Hamd17-C | RMCC | 0.0329 | 0.0044 | 0.0862 | 0.0196 |
| CTQ-PA | Hamd17-C | RdmPFC | 0.0465 | 0.0168 | 0.0956 | 0.0193 |
| CTQ-PA | Hamd17-C | LdmPFC | 0.0300 | 0.0045 | 0.0664 | 0.0155 |
| CTQ-PA | Hamd17-RD | RMCC | 0.1740 | 0.0548 | 0.3779 | 0.0782 |
| CTQ-EN | Hamd17 | RdlPFC | -0.1382 | -0.282 | -0.0328 | 0.0624 |
| CTQ-EN | Hamd17-W | RdlPFC | -0.0124 | -0.03 | -0.0016 | 0.0071 |
| CTQ-EN | Hamd17-A | RPCC | -0.0562 | -0.1139 | -0.0193 | 0.0235 |
| CTQ-EN | Hamd17-A | RvmPFC | -0.0468 | -0.0992 | -0.0115 | 0.0221 |
| CTQ-EN | Hamd17-A | LvmPFC | -0.0439 | -0.0957 | -0.0108 | 0.0208 |
| CTQ-EN | Hamd17-C | RdmPFC | -0.0248 | -0.047 | -0.0097 | 0.0094 |
| CTQ-EN | Hamd17-RD | RdlPFC | -0.0483 | -0.1146 | -0.0069 | 0.0265 |
| CTQ-PN | Hamd17 | RdlPFC | -0.2523 | -0.4901 | -0.1136 | 0.0894 |
| CTQ-PN | Hamd17-W | RdlPFC | -0.0207 | -0.0452 | -0.005 | 0.0101 |
| CTQ-PN | Hamd17-A | RdlPFC | 0.1023 | 0.0293 | 0.2133 | 0.0460 |
| CTQ-PN | Hamd17-C | RvlPFC | -0.0346 | -0.071 | -0.0127 | 0.0142 |
| CTQ-PN | Hamd17-RD | RdlPFC | -0.1938 | -0.3396 | -0.0875 | 0.0629 |
| **Posterior IFC Network** | | | | | | |
| CTQ | Hamd17 | RCun | 0.0621 | 0.0177 | 0.1318 | 0.0287 |
| CTQ | Hamd17 | RMTG | 0.0474 | 0.0126 | 0.1022 | 0.0220 |
| CTQ | Hamd17-S | RPcu | 0.0380 | 0.0165 | 0.0697 | 0.0132 |
| CTQ | Hamd17-C | RFFA | -0.0106 | -0.0191 | -0.0046 | 0.0036 |
| CTQ | Hamd17-RD | RIPL | 0.0304 | 0.0103 | 0.0627 | 0.0131 |
| CTQ-SA | Hamd17 | LPcu | -0.4227 | -0.7705 | -0.2173 | 0.1355 |
| CTQ-SA | Hamd17-W | RdlPFC | 0.0384 | 0.0131 | 0.0834 | 0.0171 |
| CTQ-SA | Hamd17-S | RITG | -0.1432 | -0.2738 | -0.049 | 0.0560 |
| CTQ-SA | Hamd17-A | LvlPFC | -0.1866 | -0.3473 | -0.0936 | 0.0621 |
| CTQ-SA | Hamd17-A | RdlPFC | -0.1567 | -0.3262 | -0.0587 | 0.0660 |
| CTQ-SA | Hamd17-C | LPcu | 0.0648 | 0.0263 | 0.1395 | 0.0277 |
| CTQ-SA | Hamd17-C | RIPL | 0.0622 | 0.0251 | 0.1283 | 0.0256 |
| CTQ-SA | Hamd17-RD | LdlPFC | 0.2903 | 0.1383 | 0.4841 | 0.0885 |
| CTQ-SA | Hamd17-RD | RdlPFC | 0.3324 | 0.1571 | 0.569 | 0.1038 |
| CTQ-SA | Hamd17-RD | LvlPFC | 0.2760 | 0.1313 | 0.5017 | 0.0900 |
| CTQ-SA | Hamd17-RD | bdmPFC | 0.2383 | 0.0847 | 0.4676 | 0.0951 |
| CTQ-SA | Hamd17-RD | LPut | 0.1961 | 0.0576 | 0.413 | 0.0885 |
| CTQ-SA | Hamd17-RD | RIns | 0.1416 | 0.0498 | 0.2928 | 0.0584 |
| CTQ-EA | Hamd17-A | RdlPFC | -0.1051 | -0.208 | -0.0364 | 0.0424 |
| CTQ-EA | Hamd17-RD | LdlPFC | 0.1183 | 0.0424 | 0.2568 | 0.0521 |
| CTQ-EA | Hamd17-RD | LdmPFC | 0.0903 | 0.0272 | 0.206 | 0.0434 |
| CTQ-EA | Hamd17-RD | RIns | 0.0873 | 0.0254 | 0.1839 | 0.0394 |
| CTQ-PA | Hamd17 | RHip | 0.2136 | 0.0258 | 0.5695 | 0.1358 |
| CTQ-PA | Hamd17-S | RHip/PHG | 0.1375 | 0.0445 | 0.275 | 0.0583 |
| CTQ-PA | Hamd17-S | LHip/PHG | 0.1288 | 0.0388 | 0.2901 | 0.0632 |
| CTQ-PA | Hamd17-A | RvlPFC | 0.1450 | 0.0613 | 0.265 | 0.0516 |
| CTQ-PA | Hamd17-A | LHip | 0.1233 | 0.0315 | 0.2926 | 0.0652 |
| CTQ-PA | Hamd17-RD | RdlPFC | -0.2183 | -0.3949 | -0.0799 | 0.0790 |
| CTQ-PA | Hamd17-RD | RvlPFC | -0.2405 | -0.4284 | -0.1066 | 0.0814 |
| CTQ-EN | Hamd17 | RPcu | 0.1302 | 0.0404 | 0.2754 | 0.0597 |
| CTQ-EN | Hamd17-S | RPcu | 0.0776 | 0.0351 | 0.1427 | 0.0268 |
| CTQ-EN | Hamd17-S | LHip/PHG | 0.0511 | 0.016 | 0.1107 | 0.0236 |
| CTQ-EN | Hamd17-A | LHip | 0.0553 | 0.0142 | 0.1257 | 0.0280 |
| CTQ-EN | Hamd17-C | LIPL | 0.0271 | 0.0098 | 0.0513 | 0.0271 |
| CTQ-EN | Hamd17-C | LMCC | 0.0292 | 0.0111 | 0.0537 | 0.0108 |
| CTQ-EN | Hamd17-C | RvlPFC | 0.0167 | 0.0045 | 0.0375 | 0.0080 |
| CTQ-EN | Hamd17-C | LIns | 0.0192 | 0.0039 | 0.043 | 0.0098 |
| CTQ-EN | Hamd17-RD | LdlPFC | 0.0986 | 0.0307 | 0.1999 | 0.0420 |
| CTQ-EN | Hamd17-RD | LIPL | 0.0949 | 0.0273 | 0.1901 | 0.0410 |
| CTQ-PN | Hamd17 | RPcu | 0.3096 | 0.1265 | 0.5874 | 0.1155 |
| CTQ-PN | Hamd17-S | RPcu | 0.1970 | 0.0984 | 0.3357 | 0.0598 |
| CTQ-PN | Hamd17-S | LHip/PHG | 0.1133 | 0.0451 | 0.224 | 0.0459 |
| CTQ-PN | Hamd17-A | RdlPFC | 0.0942 | 0.0285 | 0.2019 | 0.0433 |
| CTQ-PN | Hamd17-A | LHip | 0.1109 | 0.0475 | 0.2026 | 0.0393 |
| CTQ-PN | Hamd17-C | LdlPFC | 0.0285 | 0.0088 | 0.0644 | 0.0137 |
| CTQ-PN | Hamd17-RD | bdmPFC | -0.1289 | -0.2672 | -0.0366 | 0.0593 |

**Abbreviation:** HAMD-17, 17-items Hamilton Depression Scale; HAMD-A, HAMD-17 Anxiety/somatization subscale; HAMD-W, HAMD-17 Weight loss subscale; HAMD-C, HAMD-17 Cognitive disturbance subscale; HAMD-RD, HAMD-17 Retardation subscale; HAMD-S, HAMD-17 Sleep disruption subscale; RPcu, right precuneus; LPcu, left precuneus; LPCC, left posterior cingulate cortex; RdlPFC, right dorsal lateral prefrontal cortex; LvlPFC, left ventral lateral prefrontal cortex; RMCC, right middle cingulate cortex; LdlPFC, left dorsal lateral prefrontal cortex; RACC, right anterior cingulate cortex; LdmPFC, left dorsal medial prefrontal cortex; RCau, right caudate nucleus; LCau, left caudate nucleus; LMCC, left middle cingulate cortex; bPCC, bilateral posterior cingulate cortex; RvlPFC, right ventral lateral prefrontal cortex; LACC, left anterior cingulate cortex; RTha, right thalamus; LvmPFC, left ventral medial prefrontal cortex; RdmPFC, right dorsal medial prefrontal cortex; bACC, bilateral anterior cingulate cortex; RvmPFC, right ventral medial prefrontal cortex; LOFC, left orbitofrontal cortex; ROFC, right orbitofrontal cortex; RPut, right putamen; LIns, left insula; bPcu, bilateral precuneus; LHip, left hippocampus; LPHG, left parahippocampus; bTha, bilateral thalamus; RPCC, right posterior cingulate cortex; RCun, right cuneus; RMTG, right middle temporal gyrus; RFFA, right fusiform area; RIPL, right inferior parietal lobe; RITG, right inferior temporal gyrus; bdmPFC, bilateral dorsal medial prefrontal cortex; LPut, left putamen; RIns, right insula; RHip, right hippocampus; RHip/PHG, right hippocampus/parahippocampus; LHip/PHG, left hippocampus/parahippocampus; LIPL, left inferior parietal lobe.


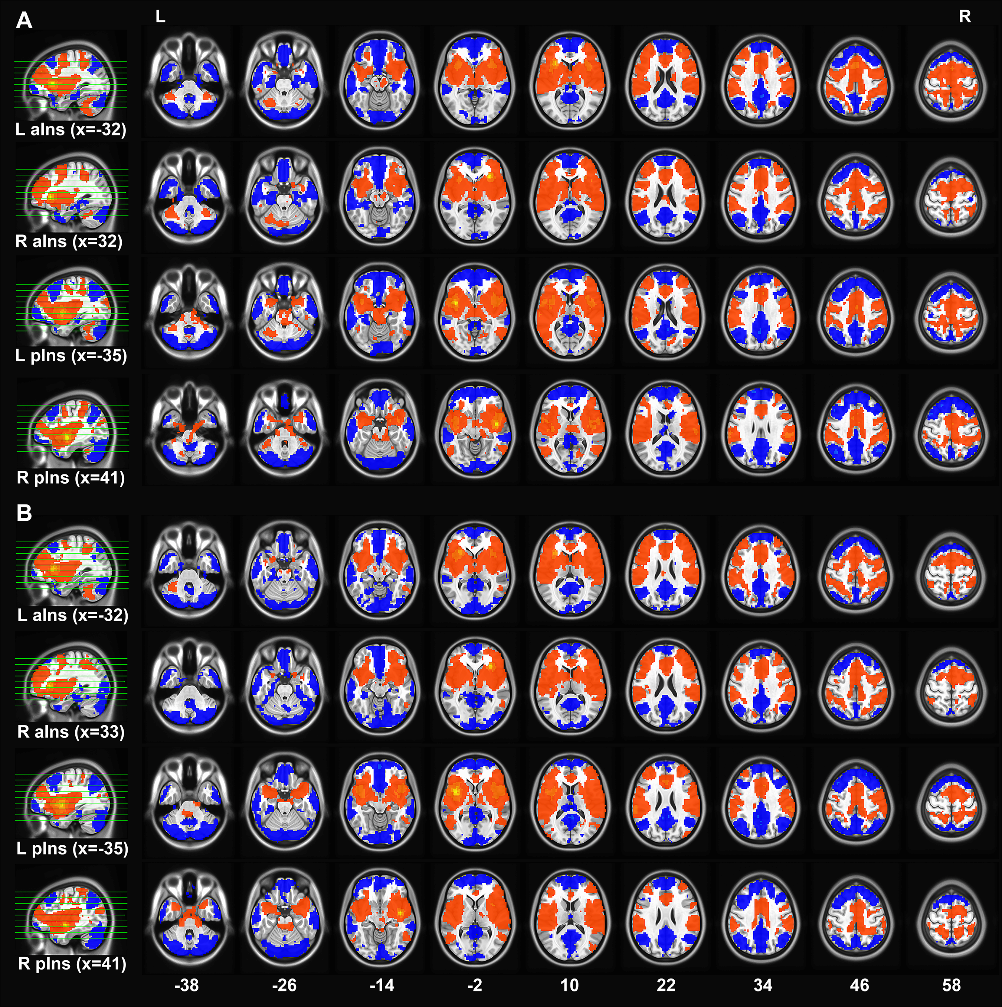


**Figure S1**. **Resting-state functional connectivity pattern of distinctive insula networks across all participates.** The results illustrate the different neural constructs of bilateral insula networks for HC (A) and MDD (B) subjects by using one sample T test. Bright color indicates positive connectivity and blue color indicates negative connectivity. Color bar is presented with Z scores. **Abbreviation:** L, left; R, right; aIns, anterior insula; pIns, posterior insula.


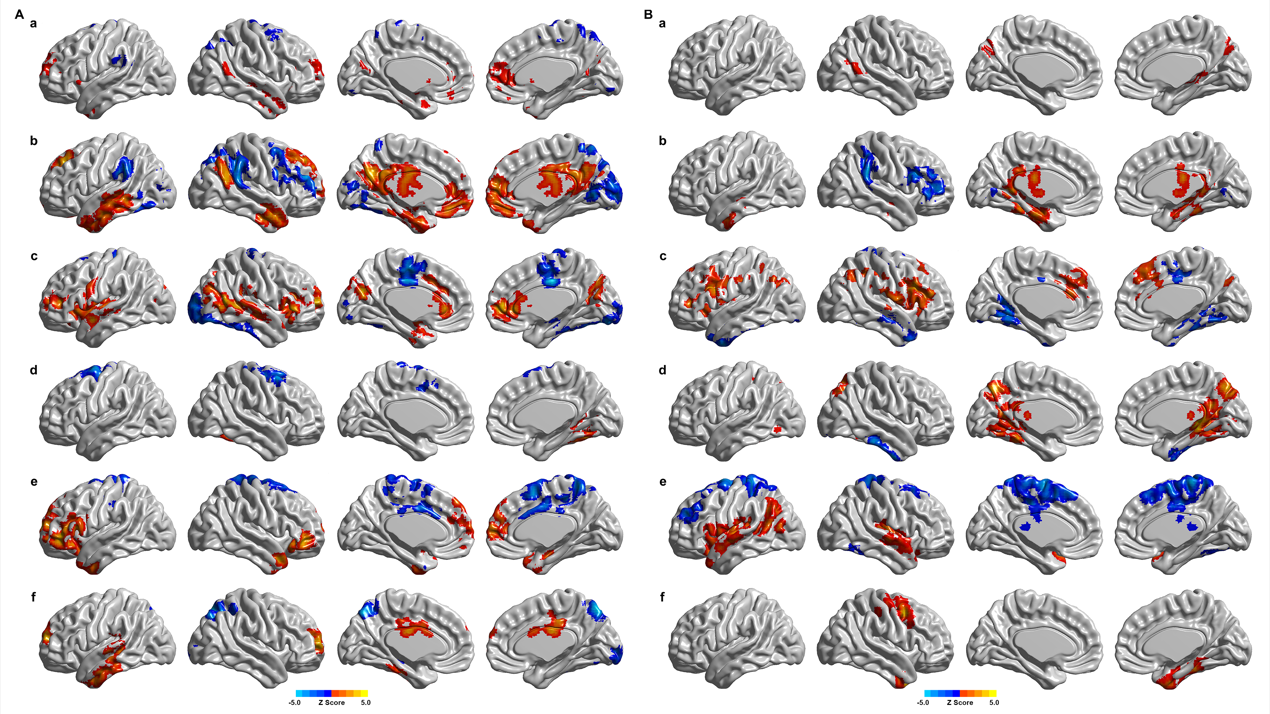


**Figure S2. Neural effects of HAMD-17 total and sub-factor scores on anterior and posterior IFC networks in MDD patients.** **A)** Main effects of HAMD-17 scores (a), HAMD-Anxiety scores (b), HAMD-Retardation scores (c), HAMD-Sleep scores (d), HAMD-Cognitive disturbance subscale scores (e), and HAMD-Weight scores (f) on the aIFC networks in the MDD patients. **B)** Main effects of HAMD-17 scores (a), HAMD-Anxiety scores (b), HAMD-Retardation scores (c), HAMD-Sleep scores (d), HAMD-Cognitive disturbance subscale scores; (e), and HAMD-Weight scores (f) on the pIFC networks in the MDD patients. Red color indicates positive correlation and blue color indicates negative correlation. **Abbreviation:** HAMD-17, 17-items Hamilton Depression Scale; HAMD-Anxiety, HAMD-17 Anxiety/somatization subscale; HAMD-Weight, HAMD-17 Weight loss subscale; HAMD-Cognition, HAMD-17 Cognitive disturbance subscale; HAMD-Retardation, HAMD-17 Retardation subscale; HAMD-Sleep, HAMD-17 Sleep disruption subscale; aIFC, anterior insula functional connectivity; pIFC, posterior insula functional connectivity.
